# Pregnancy and contraceptive use among participants of childbearing potential in the HVTN 705 HIV vaccine trial in Southern Africa

**DOI:** 10.3389/frph.2025.1565933

**Published:** 2025-06-10

**Authors:** Pamela Mda, Kathryn Mngadi, Bo Zhang, Randy Burnham, Michal Juraska, Ollivier Hyrien, Nigel Garrett, Thozama Dubula, Sinalo Toni, Sibi Joseph, Phillip Kotze, Susan Buchbinder, Azwidihwi Takalani, Frank Tomaka, Alexander Luedtke, Wouter Willems, Edith Swann, Julia Hutter, Huub Gelderblom, M. Juliana McElrath, Ludo Lavreys, Lynda Stranix-Chibanda, Alison C. Roxby, Linda-Gail Bekker, Glenda E. Gray

**Affiliations:** ^1^ Nelson Mandela Academic Clinical Research Unit, Walter Sisulu University, Mthatha, South Africa; ^2^ The Aurum Institute, Johannesburg, South Africa; ^3^ Vaccine and Infectious Diseases Division, Fred Hutchinson Cancer Center, Seattle, WA, United States; ^4^ Centre for the AIDS Programme of Research in South Africa, University of KwaZulu–Natal, Durban, South Africa; ^5^ Desmond Tutu HIV Centre, Cape Town, South Africa; ^6^ Qhakaza Mbokodo Research Clinic, Ladysmith, South Africa; ^7^ San Francisco Department of Public Health, San Francisco, CA, United States; ^8^ Department of Medicine, University of California, San Francisco, San Francisco, CA, United States; ^9^ Hutchinson Center Research Institute of South Africa, Chris Hani Baragwanath Academic Hospital, Soweto, South Africa; ^10^ Janssen Research & Development, Titusville, NJ, United States; ^11^ Janssen Research & Development, Beerse, Belgium; ^12^ Division of AIDS, National Institute of Allergy and Infectious Diseases, Bethesda, MD, United States; ^13^ Clinical Trials Research Centre, University of Zimbabwe, Harare, Zimbabwe; ^14^ Departments of Medicine and Global Health, University of Washington, Seattle, WA, United States; ^15^ Department of Medicine, University of Cape Town, Cape Town, South Africa; ^16^ South African Medical Research Council, Pretoria, South Africa

**Keywords:** HIV-1 vaccine trials, HIV prevention, HIV incidence, contraception, pregnancy

## Abstract

**Background:**

HIV vaccine trial participants include sexually active cisgender females who agree to avoid pregnancy during the active vaccination period. Nevertheless, some pregnancies occur in almost all studies. We examined contraceptive use, pregnancy incidence, and the relationship between pregnancy and HIV seroconversion in one HIV vaccine trial.

**Methods:**

We performed an exploratory analysis of data collected for HVTN 705/HPX2008, a phase IIb HIV vaccine trial enrolling cisgender women across 23 sites in five southern African countries. Baseline characteristics and contraceptive use were assessed among participants who became pregnant and those who did not during the active vaccination phase (months 0–15). Pregnancy incidence rates were calculated for this phase and the duration of follow up (36 months). Cox regression analysis was used to assess factors associated with incident pregnancy.

**Results:**

There were 2,636 participants who received at least one vaccine or placebo dose (mean age: 23 years, standard deviation: 3 years). At enrolment, when contraception was required, 62.9% reported using injectable contraceptives. Overall pregnancy rate was 2.95 per 100 person-years (95% CI: 2.40, 3.58), with 101 pregnancies reported by month 15. Cumulative incidence of pregnancy at month 15 was similar between trial arms (log-rank *p* = 0.688). Each additional year of age was associated with an 8% decrease in pregnancy incidence (*p* = 0.014). Women aged 31–35 years had the lowest pregnancy incidence [1.75 (0.48, 4.48) per 100 person-years]. In a Cox regression analysis covering months 0–15, all contraceptive methods significantly reduced the incidence of pregnancy compared to no contraceptive use. Oral contraception was associated with the least reduction in pregnancy risk; implants were associated with the most reduction in pregnancy risk (*p* < 0.001).

**Conclusions:**

In HVTN 705/HPX2008, higher incidence of pregnancy was associated with younger age and oral contraception (compared to other methods). These data may inform future designs of HIV prevention or vaccine trials.

## Introduction

Per regulations, experimental vaccine products are tested among healthy adults first, with inclusion of pregnant persons only once vaccine safety has been determined (1). Participants of childbearing potential are required to use contraception to meet study eligibility criteria, and are provided detailed information during the informed consent process to enter a vaccine trial, however, pregnancies still occur (2, 3). In southern Africa, adolescent girls and young women (ages 15 to 24) are three times more likely to acquire HIV than adolescent boys and young men of the same age (4). Clinical trials are often focused on women during their years of childbearing potential due to the high incidence of HIV in this group (5).

For those requested to avoid pregnancy in clinical trials, multiple contraceptive methods are available, and the method mix varies by country, region, and access to care. In southern Africa, injectables account for 33% of all contraceptive usage, making them the predominant method, a distinction not seen in any other region (6). Longer-acting contraceptive methods such as intrauterine devices (IUDs), injectables and implants are not user dependent and are the most effective in typical use; however, requiring specific contraceptives and denying contraceptive choice for trial participants is challenging to implement, as each method can have different effects on the user (7). Asking potential trial participants to switch contraceptive methods when they enter a trial can adversely affect participants, especially for those who are satisfied with their current method. Contraceptive consistency is important and most clinicians avoid asking users to discontinue a method that they are accustomed to (8). For these reasons, most clinical trials allow participants to choose their own method if contraceptive type is unlikely to influence trial results. Some studies provide access to contraception on site, which is preferable (9), but this is not always feasible.

A further complication of recent HIV clinical trials are the long duration of studies. The process of stimulating the creation of HIV-specific antibodies through vaccination is currently thought to require extended vaccination regimens, involving sequential immunizations to prime a sequence of events to develop neutralizing anti-HIV antibodies (10, 11). Some proposed administration schedules for vaccines can span beyond 12 months in duration. For participants of childbearing potential, these extended regimens can represent a significant period of time when trial participation is requesting the use of contraception and the avoidance of pregnancy (2, 8). Over such a long period of time, women may tire of their contraception, struggle with adherence, or they may have adverse experiences with contraception and decide to change methods. Partnerships may dissolve or form and participants' desire for pregnancy may evolve or change, Further, women may not have complete autonomy over the decision to use or discontinue contraception.

In this era of complex vaccination regimens against HIV, it is important to understand the experiences of women using contraception as they navigate the requirement to avoid pregnancy while participating in a lengthy vaccine trial. Our aim was to describe the patterns of contraceptive use, evaluate factors associated with incident pregnancy, and describe the relationship between incident pregnancy and HIV acquisition among participants in the phase 2b randomized placebo-controlled trial of Ad26.Mos4.HIV and clade C gp140 immunogens (HVTN 705/HPX2008, NCT03060629).

## Methods

This was a secondary analysis of data from a multicenter, randomized, double-blind, placebo-controlled phase 2b efficacy study of a heterologous vaccine regimen of tetravalent Ad26.Mos.HIV and Aluminum Phosphate-adjuvanted Clade C gp140 in preventing HIV-1 acquisition among women in sub-Saharan Africa (HVTN 705/HPX2008), otherwise known as the Imbokodo study.

Participants received 4 injections at months 0, 3, 6 and 12. At months 0 and 3, participants received Ad26.Mos.HIV or placebo, and at months 6 and 12 participants received Ad26.Mos.HIV and Aluminum Phosphate-adjuvanted Clade C gp140 or placebo. The primary endpoint of the study was diagnosis of HIV-1 infection. The study was conducted at 23 research sites in 5 African countries: Malawi, Mozambique, South Africa, Zambia and Zimbabwe. Participants were recruited from populations with high HIV incidence between November 2017 and June 2019.

Enrolled participants were sexually active cisgender females between the ages of 18 and 35 years living without HIV. Participants were randomly assigned in a 1:1 ratio to either the vaccine or placebo group. The study was discontinued in August 2021 because there was no evidence of vaccine efficacy at an interim analysis. Further details of the parent study including methods, eligibility criteria, and results are described elsewhere (12).

As part of study participation, women of childbearing potential agreed to use the effective contraception method of their choice from 21 days prior to receiving the first dose of investigational product or placebo (month 0) through 3 months after the last injection at week 48. Contraception use would thus last through month 15 for participants who received their vaccinations according to the protocol schema. During screening, women who were not already using contraception were asked to begin use at least 21 days before enrollment. Women were asked at each study visit to report the contraception that they were using, which was then captured on a pregnancy prevention case report form. If possible, this was corroborated with clinic records or contraception cards. Some, but not all, study sites offered contraception at the same location. Contraception method switches were recorded. Pregnancy testing was performed at the screening visit and at months 0, 3, 6, 12 and 33, except for participants who had medical records confirming total hysterectomy, bilateral oophorectomy, premature menopause, and/or bilateral tubal ligation; these participants were considered to lack reproductive potential. When a participant became pregnant during the study, subsequent vaccinations were discontinued. If that participant was no longer pregnant and willing to continue participating, vaccination was resumed if the participant was within a vaccination window. Our analyses included every participant's first study pregnancy reported in the first 15 months as well as all first pregnancies during the entire study period. Therefore, this analysis includes more pregnancies than reported in the efficacy trial results, which were limited to pregnancies within 3 months of vaccinations, defined as week 0 to week 48 plus an additional 3 months.

### Statistical analysis

The analysis used data from the primary study cohort Full Analysis Set (FAS) population and included all randomized participants who received at least 1 investigational product administration. Baseline characteristics including age, socioeconomic status, country of residence, and choice of contraceptives of the study population were reported. Differences in these factors among participants with a reported pregnancy between month 0 and the month 15 visit and participants who did not report a pregnancy during the same time period were assessed. The same descriptive analysis was reported for the period between month 0 and month 36. Kaplan–Meier curves stratified by vaccine and placebo arms were displayed and a log-rank test was used to assess the difference in the rate of reported pregnancy in the vaccine group vs. placebo group (1) between month 0 to month 15 (period of active vaccination + 3 months); (2) between month 0 to month 24; and (3) between month 0 to month 36.

A prespecified primary analysis used the Cox proportional hazards model to assess factors, including the most recent recorded contraceptive method modelled as a time-varying covariate (including method reported at each study visit), associated with cumulative pregnancy incidence in the primary study cohort between month 0 to month 15. Analogous Cox analyses were conducted in the primary study cohort between month 0 to month 24 and between month 0 and month 36. An exploratory analysis also examined the Per-Protocol (PP) population between month 0 and month 15, which included participants who remained without HIV 4 weeks after the 3rd vaccination visit, received all planned injections at the first 3 injection visits within the respective visit windows, and had no other major protocol deviations judged to possibly impact the efficacy of the vaccine. An exploratory Cox analysis investigated the relationship between incident pregnancy and HIV acquisition. All model terms were pre-specified. Analyses were performed with the statistical computing software R version 4.0 and/or SAS version 9.4. Statistical testing was 2-sided and *p* < .05 was considered statistically significant. Primary analyses were all pre-specified.

### Ethics

The parent trial was approved by all relevant institutional review boards and applicable regulatory entities. All participants gave written informed consent in their preferred language.

## Results

### Baseline characteristics and choice of contraception

At enrollment, of 2,636 participants, 1,658 (62.9%) reported using injectable contraceptives, 582 (22.1%) implants, 195 (7.4%) multiple or other methods, 151 (5.7%) oral contraceptives, 40 (1.5%) used IUD and 10 (0.4%) reported prior sterilization (Table 1). Contraceptive choice was highly dependent on participant location. At enrollment, South African women were more likely to use injectable contraception (72.0%) compared to women in Malawi (53.5%), Mozambique (31.1%), Zambia (48.3%) and Zimbabwe (37.5%).

**Table 1:** Baseline participant characteristics, and characteristics of participants with pregnancy by month 15 and month 36. Population: full analysis set (*N* = 2,636).

Characteristic	Month 0	Cumulative pregnancy by Month 15	Cumulative pregnancy by Month 36	No reported pregnancy
Total Enrolled	2,636	101	408	2,228
Age (years)				
Median age (Min, max)	23 (18–35)	22 (18–33)	22 (18–34)	23 (18–35)
18–20	676 (25.6%)	20 (19.8%)	104 (25.5%)	572 (25.7%)
21–30	1,788 (67.8%)	77 (76.2%)	286 (70.1%)	1,502 (67.4%)
31–35	172 (6.5%)	4 (4.0%)	18 (4.4%)	154 (6.9%)
Dwelling type^a^				
Formal	2,270 (86.1%)	93 (92.1%)	363 (89.0%)	1,907 (85.6%)
Informal	322 (13.9%)	8 (7.9%)	45 (11.0%)	287 (14.4%)
Type of living area				
Urban	2,155 (81.8%)	82 (81.2%)	343 (84.1%)	1,812 (81.3%)
Rural	447 (17.0%)	19 (18.8%)	65 (15.9%)	382 (17.1%)
Medical Aid^b^	37 (1.4%)	2 (2.0%)	4 (1.0%)	33 (1.5%)
Educational Level				
No formal education	10 (0.4%)	1 (1.0%)	3 (0.7%)	7 (0.3%)
Primary education	1,351 (51.3%)	56 (55.4%)	225 (55.1%)	1,126 (50.5%)
Secondary education	1,257 (47.7%)	43 (42.6%)	176 (43.1%)	1,081 (48.5%)
Tertiary education and above	18 (0.7%)	1 (1.0%)	4 (0.9%)	14 (0.6%)
Country of Residence				
Malawi	157 (6.0%)	8 (7.9%)	26 (6.4%)	131 (5.9%)
Mozambique	45 (1.7%)	2 (2.0%)	12 (2.9%)	33 (1.5%)
South Africa	1,774 (67.3%)	48 (47.5%)	210 (51.5%)	1,564 (70.2%)
Zambia	329 (12.5%)	29 (28.7%)	91 (22.3%)	238 (10.7%)
Zimbabwe	331 (12.6%)	14 (13.9%)	69 (16.9%)	262 (11.8%)
Had a Partner Living with HIV	51 (1.9%)	3 (3.0%)	12 (2.9%)	39 (1.8%)
Any STI^c^	844 (32.0%)	34 (33.7%)	136 (33.3%)	708 (31.8%)
Syphilis^d^	82 (3.1%)	2 (2.0%)	9 (2.2%)	73 (3.3%)
Trichomonas^e^	234 (8.9%)	13 (12.9%)	41 (10.0%)	193 (8.7%)
N. gonorrhea^f^	183 (6.9%)	5 (5.0%)	28 (6.9%)	155 (7.0%)
C. trachomatis^f^	550 (20.9%)	23 (22.8%)	81 (19.9%)	469 (21.1%)
Pregnancy prevention method^g^				
Intrauterine device (IUD) or system (IUS)	40 (1.5%)	1 (1.0%)	5 (1.2%)	35 (1.6%)
Implants	582 (22.1%)	23 (22.8%)	124 (30.4%)	458 (20.6%)
Injectable contraceptives	1,658 (62.9%)	43 (42.6%)	204 (50.0%)	1,454 (65.3%)
Oral contraceptives	151 (5.7%)	26 (25.7%)	47 (11.5%)	104 (4.7%)
Sterilization^h^	10 (0.4%)	0 (0.0%)	0 (0.0%)	10 (0.4%)
Multiple^i^, Other^j^	195 (7.4%)	8 (7.9%)	28 (6.9%)	167 (7.5%)
No. of live births at study entry				
0	546 (20.7%)	29 (28.7%)	95 (23.3%)	451 (20.2%)
1	1,272 (48.3%)	46 (45.5%)	205 (50.2%)	1,067 (47.9%)
2 or more	784 (29.7%)	26 (25.7%)	108 (26.5%)	676 (30.3%)

Full analysis set includes all participants who received at least 1 study product administration.

Includes only first pregnancies during the study for participants.

^a^ Dwelling type was missing for 34 participants. Informal housing included shacks/shanties, traditional huts, caravan, and tent.

^b^ Covered by private health insurance.

^c^ Indicates positivity for any STIs for which a participant was tested.

^d^ Both non-treponemal and treponemal test must be positive for a positive diagnosis.

^e^ Test performed on cervical/vaginal swab.

^f^ Test performed on cervical/vaginal swab or urine, result is positive if either test is positive. Some counts may not total to the number of participants, if missing.

^g^ All volunteers must agree to consistently use effective contraception per study inclusion criteria for sexual activity that could lead to pregnancy from at least 21 days prior to enrollment/first study product receipt through 3 months after the last vaccination (month 15).

^h^ Sterilization (tubal ligation/hysterectomy/laparoscopy/other medical condition that causes sterilization).

^i^ “Multiple” refers to use of more than one contraceptive method.

^j^ “Other” refers to contraceptive method other than implants, injectable, sterilization.

During the first 15 months, contraceptive patterns changed, with a gradual decline in implants (582/2636, 22.1% to 406/2250, 18.0%) and injectables (1658/2636, 62.9% to 1209/2250, 53.7%) from enrollment to month 15. There was a small increase in oral contraceptive use from 5.7% at enrollment to 9.3% (208/2250) at month 15. Overall, 2060/2250, 91.6% of participants reported using highly effective methods at month 15.

### Cumulative pregnancy incidence between trial arms

**Figure 1:**
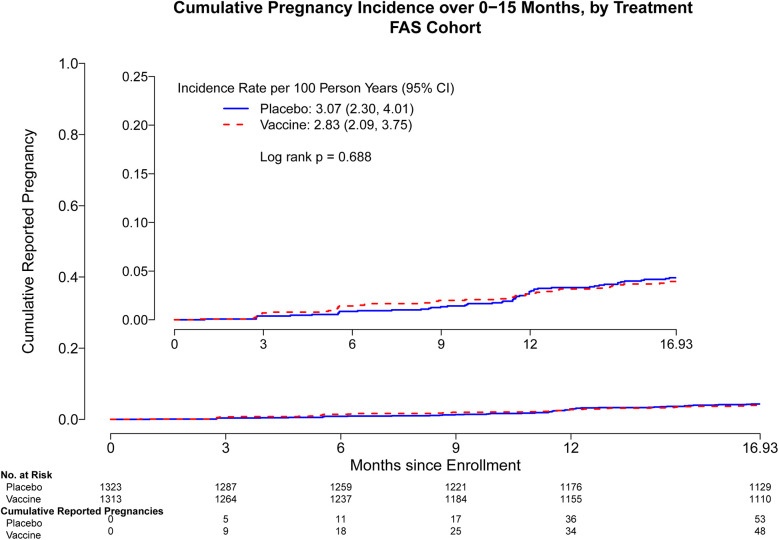
Cumulative reported pregnancy incidence, months 0–15, among all participants who received at least one study product administration, stratified by vaccine vs. placebo. Data was censored at 16.93 months which includes follow up time for participants who came late for the Month 15 visit. FAS, full analysis set, including all participants who received at least one study product administration.

**Figure 2:**
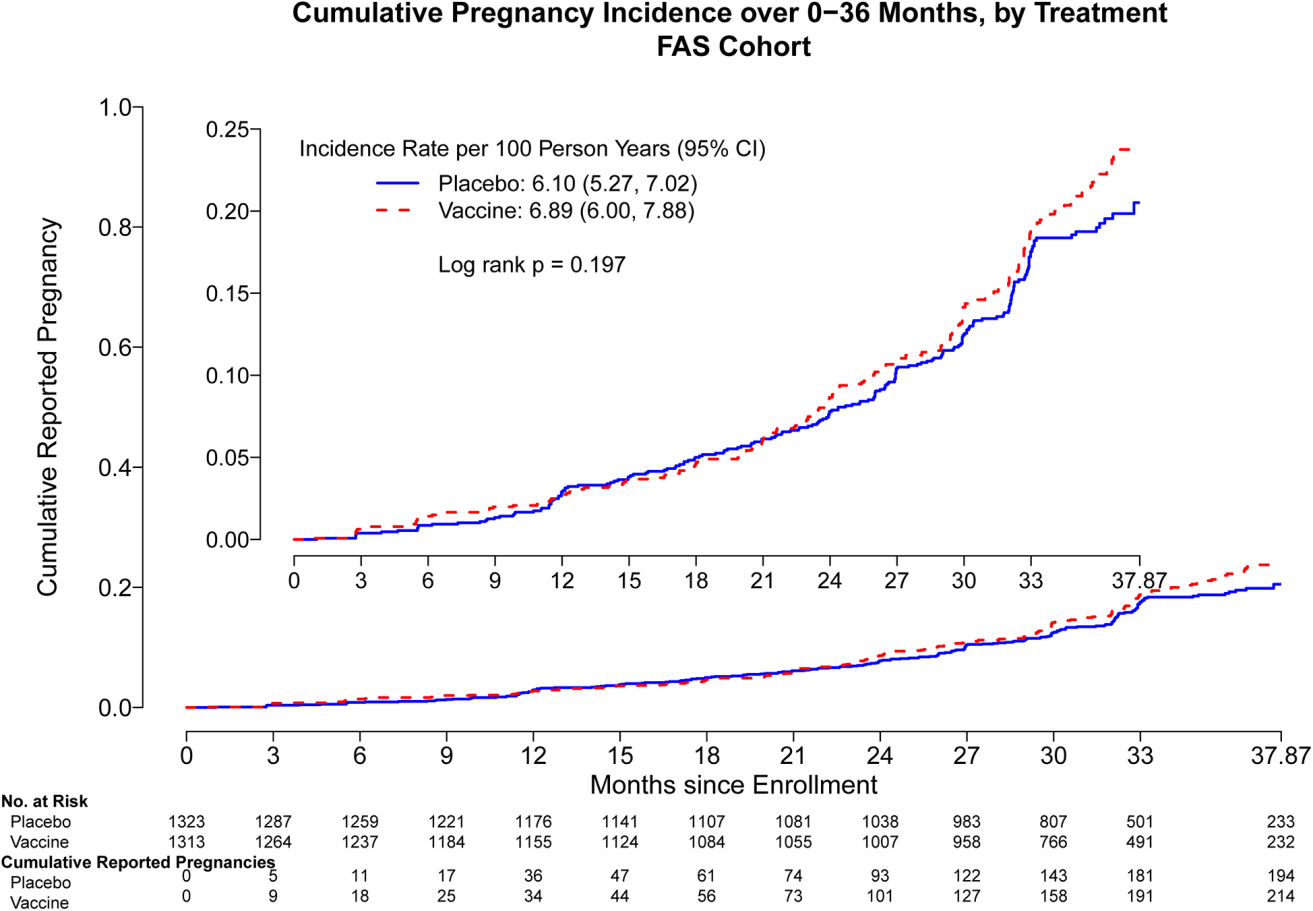
Cumulative reported pregnancy incidence, months 0–36, vaccine vs. placebo among all participants who received at least one study product administration. Data were censored at 37.87 months which includes follow up time for participants who came late for the Month 36 visit. FAS, full analysis set, including all participants who received at least one study product administration.

Through month 15, the overall pregnancy rate was 2.95 [95% confidence interval (CI): 2.40, 3.58] per 100 person-years (py) with 101 pregnancies observed. Cumulative pregnancy incidence from month 0 through month 15 did not differ by trial arm (placebo arm 3.07 per 100 py, vaccine arm 2.83 per 100 py, log-rank *p* = 0.69) (Figure 1). The incidence increased more rapidly in both arms after month 15, and there remained no difference in the pregnancy incidence between month 0 and month 24 (placebo arm 3.99 per 100 py, vaccine arm 4.64 per 100 py, log-rank *p* = 0.26) (Supplementary Figure S1) and between month 0 and month 36 (placebo arm 6.10 per 100 py, vaccine arm 6.89 per 100 py, log-rank *p* = 0.19) (Figure 2). Analyses were repeated in the per-protocol cohort, and the results were similar (placebo arm 5.7 per 100 py, vaccine arm 6.08 per 100 py, log-rank *p* = 0.53) (Supplementary Figure S2).

### Factors associated with incident pregnancy during the first 15 months

**Figure 3:**
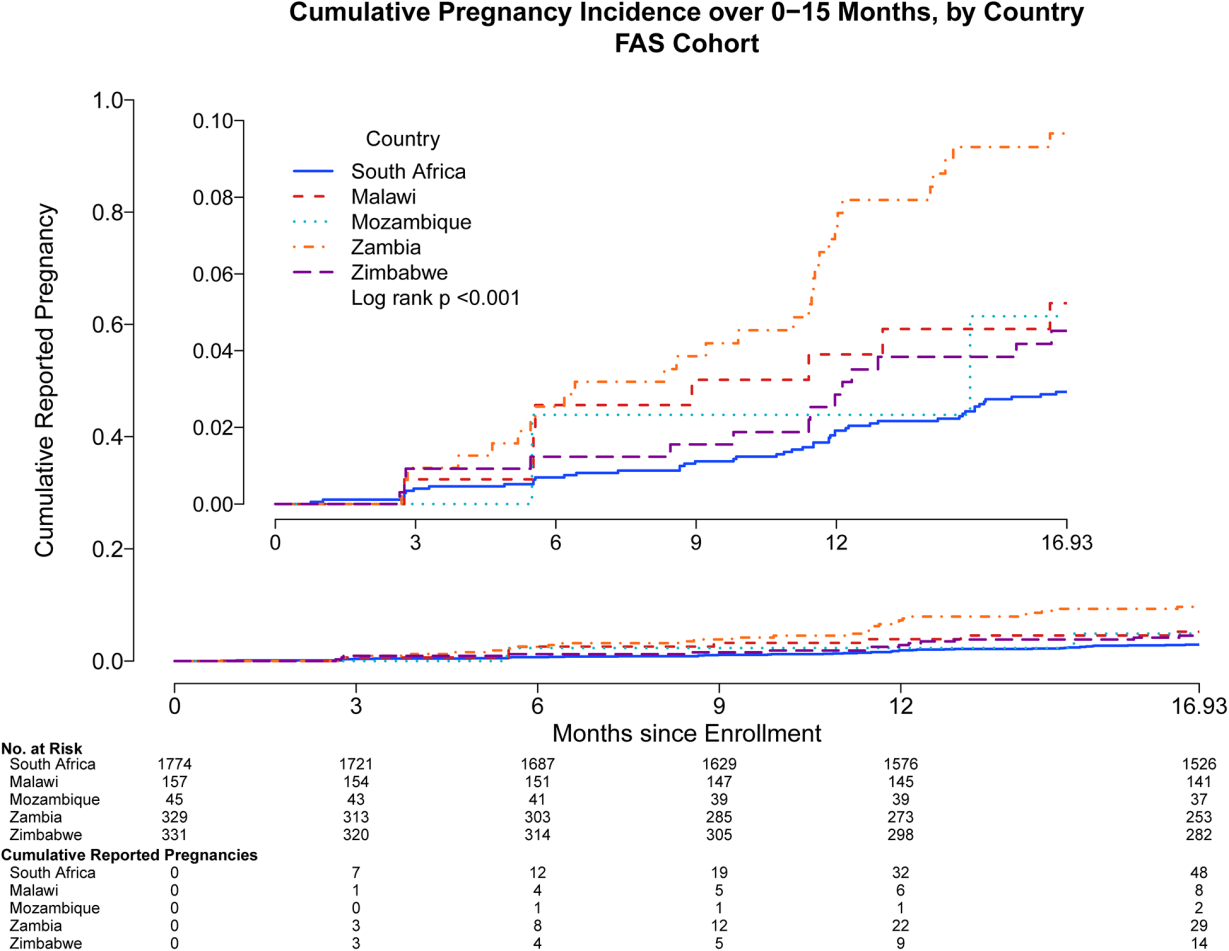
Cumulative reported pregnancy incidence, months 0–15, among all participants who received at least one study product administration, stratified by country of enrollment. Data were censored at 16.93 months which includes follow up time for participants who came late for the Month 15 visit. Full analysis set includes all participants who received at least one study product administration.

Pregnancy incidence rates reported through month 15 were different among participating countries: Zambia reported 29 pregnancies [7.09 95% CI: (4.75, 10.19) per 100 py], Malawi 8 [3.81 95% CI: (1.65, 7.51) per 100 py], Mozambique 2 [3.52 95% CI: (0.43, 12.73) per 100 py], Zimbabwe 14 [3.24 95% CI: (1.77, 5.43) per 100 py], and South Africa 48 [2.07 95% CI: (1.53, 2.75) per 100 py] (Figure 3). Most pregnancies occurred among those between the ages of 21–30 years [77; 3.33 95% CI: (2.63, 4.16) per 100 py], with fewer for adolescents aged 18–20 years [20; 2.27 95% CI: (1.39, 3.50) per 100 py], and the fewest pregnancies among women between 31 and 35 years [4; 1.75 95% CI: (0.48, 4.48) per 100 py].

Users of different contraceptive methods had different pregnancy incidence between month 0 to 15 (oral pills: 14.61 95% CI: [9.54, 21.41] per 100 py; implants: 2.99 95% CI: [1.90, 4.49] per 100 py; injectables: 1.99 95% CI: [1.44, 2.68] per 100 py; other: 2.85 95% CI: [1.30, 5.41] per 100 py; log rank *p* < 0.001) and oral contraceptive users had the highest cumulative incidence of pregnancy (Supplementary Figure S3). No difference in socio-economic status, partner's HIV status, or baseline sexually transmitted infections was observed among women pregnant through month 15 and those not pregnant (data not shown).

At month 15, which for most participants was three months after their last study injection, participants were no longer asked to use contraception. During this later period of the study (month 15–36) the cumulative pregnancy incidence increased more rapidly, and an additional 307 women had a first study pregnancy.

### Factors associated with incident pregnancy over 36 months

Overall, participants who became pregnant during months 0–36 were more likely to be living in an urban area (chi-squared test *p* = 0.03), reported using oral contraceptives (*p* < 0.001), and most likely from countries outside South Africa (*p* < 0.001) (Supplementary Table S1).

Incidence rates of pregnancy for the entire study were more than double the rates observed during the first 15 months. A Cox regression analysis of factors associated with incident pregnancy from months 0–15 and accounting for time-varying contraceptive methods found that, compared to no contraceptive use, each contraceptive method was associated with significant pregnancy prevention, with the implant being the most effective and reducing the pregnancy incidence by 99.6% [HR = 0.004; 95% CI: (0.002, 0.012), *p*-value < .001], followed by injectables reducing the incidence by 99.0% [HR = 0.010; 95% CI: (0.006, 0.018), *p*-value < 0.001]. Oral contraceptives were the least effective, reducing the incidence by 89.5% [HR = 0.105; 95% CI: (0.059, 0.188), *p*-value < 0.001]. Age was also found to be associated with pregnancy risk, with 8% decrease in pregnancy incidence for every additional year of age [HR = 0.920; 95% CI: (0.860, 0.984), *p*-value = 0.014]. Different levels of education were not associated with pregnancy (HR 0.731–1.704) (Table 2).

**Table 2:** Multivariate Cox proportional hazards model of characteristics associated with pregnancy in HVTN 705, among all participants who received at least one immunization.

Covariates	Adjusted Hazard Ratio (aHR)	95% CI	*P*-value
Age (per year)	0.920	0.860, 0.984	0.014
Contraceptive method			
No contraception	*Reference*		
Implants	0.004	0.002, 0.012	<0.001
Injectables	0.010	0.006, 0.018	<0.001
Oral	0.105	0.059, 0.188	<0.001
Other contraceptive	0.037	0.016, 0.084	<0.001
Education			
Secondary education or greater	*Reference*		
No formal education	0.00	0.00—inf	0.995
Some primary education	1.117	0.477, 2.615	0.799
Completed primary education only	1.704	0.743, 3.911	0.208
Completed primary and some secondary education	0.731	0.440, 1.214	0.226

CI, confidence interval. Contraceptive methods were time-varying as reported by participants at each study visit. “Other contraceptive” includes IUD, external condoms, internal condoms, diaphragm, patch, sterilization, menopause. The model evaluated data with right-censoring.

### HIV incidence by pregnancy status

During the study, a total of 8 participants reported both pregnancy and a diagnosis of HIV- 1 acquisition. Out of the 8 participants; 4 (50%) were diagnosed with HIV-1 before reporting pregnancy, while the other 4 (50%) reported pregnancy before acquiring HIV-1. None of these 8 participants were diagnosed with HIV-1 during month 0–15.

### Birth outcomes

Pregnancy outcomes were reported in the primary manuscript. There were 31 pregnancies among women within 3 months of receiving study product. No congenital anomalies were noted among the children of 24 of the women; the other 7 infants were not able to be assessed.

## Discussion

Our analysis provides valuable insights into contraceptive patterns and incident pregnancy rates among participants enrolled in a large efficacy trial. Injectable contraceptives were by far the most popular method at the time of study entry, and almost a quarter of women were using implants. However, over time, increasing proportions of women were switching contraceptives. Data to establish the motivation for switching were not available. Method choice varied significantly depending on the location and presumably the local method mix that was available. It is not surprising that the lowest pregnancy rates were observed among South African women, who were also most likely to use injectable contraceptives, particularly long-acting injectables. In other southern African settings such as Mozambique and Zimbabwe, oral contraceptives were more commonly used and pregnancies occurred more frequently. These regional differences could be associated with local factors and regional healthcare practices, as well as different baseline fertility rates and fertility intentions in different countries (13–15).

The observed pregnancy rates of 2.95 per 100 py were comparable or lower than those of other vaccine phase I and II trials (including HIV vaccine trials) conducted in this region, which reported similar pregnancy rates between 3.09 and 6.80 per 100 py (2, 3). The pregnancy rate is also lower compared to other non-vaccine HIV prevention trials in the region, where a rate of 3.95 per 100 py was observed despite onsite provision of contraceptives to the participants as well as a comprehensive contraceptive curriculum (14, 16). Other HIV prevention trials have noted similar pregnancy rates. In the Antibody Mediated Prevention (AMP) efficacy study HVTN 703/HPTN 081, where dual contraceptive use was required, there was a notable difference between incident pregnancy in participants who were provided contraceptives onsite, compared to those who were not (17). Also, some of the microbicide HIV prevention trials demonstrated increased pregnancy rates of 10.8 per 100 py and 13.4 per 100 py in MDP 301 and VOICE/MTN-003 respectively (18, 19). The contraception patterns in MDP and VOICE were comparable to those in our study, with the use of injectables being the most preferred method in South Africa.

Our finding that oral contraceptive use was associated with more pregnancies is consistent with longstanding evidence that oral contraceptives in typical use are are known to be less effective than longer-acting reversible contraception (LARC), due to many factors including possible adherence challenges, cost, and the need to resupply the method each month. There may also be differences in the fertility desires of women choosing oral contraceptives, because of shorter return to fertility than is possible using longer-acting methods (20). While it is known that oral contraceptives have lower effectiveness than longer-acting methods, there are reasons to continue to offer them as part of the method mix for trial participants. First, for most participants, they are very effective. Second, oral contraceptives have a different side effect profile than longer-acting methods, and have different effects on menstruation (20, 21). Third, oral contraceptives are the most easily reversed of the other common methods here and can be reversed under user control. In contrast, implants and IUDs require a medical visit to discontinue, and progestin-analogue injectable contraceptives are known to affect fertility for several months after cessation, especially among those who have used this method for more than a year (22). Requiring trial participants to use only certain types of contraception unfairly limits choice and may result in women not wanting to participate in trials if they are successfully using oral contraception or do not desire the effects of some of the longer acting methods.

A marked increase in pregnancy rates was observed after the 15 month timepoint (end of active vaccination), demonstrating that participants were using contraception and postponing pregnancy as was required as part of study participation. Despite agreeing to postpone pregnancy during the active vaccination period, women in this study appeared to have additional fertility desires reflected in the increased pregnancy rates after vaccination was over. However, it is not known whether the pregnancies that occurred in this study were intended or unintended. Other HIV prevention trials have observed a consistent correlation between young age and high incidence of pregnancy, which was aligned with our study results (15, 23).

Although pregnancy has been associated with increased risk of HIV acquisition (24), few seroconversions occurred among women with a pregnancy, and these seroconversions were evenly split before and after pregnancy. With these small numbers we lacked power to assess any increased risk of HIV acquisition.

Our findings suggest that this trial was able to successfully recruit cisgender women who were able to postpone fertility during trial participation. Going forward, enhanced contraceptive counselling and other strategies to ensure contraceptive success are recommended in early phase trials, especially among younger participants. For example, providing access to highly effective contraception coupled with contraceptive counselling at the study clinic has been shown to be associated with improved adherence to contraception, and is linked to better ability to monitor contraception use, as was seen in CAPRISA 004 (25). Risk scoring has also been developed to predict participants who would benefit from additional contraceptive counselling (26).

A meta-analysis of 38 HIV prevention trials identified that pregnancies occurred in 8% of participants, despite contraceptive use requirements (9). Study designs need to accommodate the reproductive desires of women participating for extended periods in HIV vaccine research, especially considering long and complex vaccination regimens. To accommodate participants' fertility desires, some feasible changes can be made to trial design. Enrolling enough participants to accommodate early study discontinuation for those who desire pregnancy is a modification that may ensure that participants can leave the study without impairing the ability of the study to reach endpoints and without creating a large group of under-vaccinated participants who do not complete the study regimen. Vaccine trials have also been constructed on calendar time intervals with monthly or quarterly visits or injection schedules; careful assessment of data could show that similar immunogenicity can be achieved with shorter intervals between vaccine doses, which would reduce the amount of time that participants need to avoid pregnancy.

Our study had limitations. Participants’ self-report was used to verify contraceptive methods and adherence. Some but not all sites dispensed contraceptives directly to participants, leading to uneven ascertainment of actual contraceptive use. Contraceptive method was strongly associated with country of residence of the participant, which can result in confounding. Results from this study may not apply to vaccine trial participants in other settings. Some pregnancies may have occurred between visits and may not have been recorded if the pregnancy did not continue.

In summary, this study highlights the importance of effective contraception in preventing pregnancies during the period around study injections. Despite a requirement to use effective contraception during the vaccination period, pregnancies occurred. Fertility was highest during the follow up period when contraception use was not required. Future HIV vaccine trials should be designed to consider that women's desire to use contraception may change during periods of extended follow up.

## Data Availability

Publicly available datasets were analyzed in this study. This data can be found here: https://atlas.scharp.org/project/HVTN%20Public%20Data/begin.view.
