# Supplementary material for: Pregnancy and contraceptive use among participants of childbearing potential in the HVTN 705 HIV vaccine trial in Southern Africa
Source: Front Reprod Health. 2025 Jun 10;7:1565933. doi: 10.3389/frph.2025.1565933 (PMC12185537; doi:10.3389/frph.2025.1565933)
Supplement: Supplementary file 1 [file Datasheet1.docx]

SUPPLEMENTARY TABLES AND FIGURES

**Table S1: Factors associated with incident pregnancy over 36 months.**

| Characteristic | Pregnant by Month 36 (N=408) | No Reported Pregnancy (N=2228) | P value^1^ |
| --- | --- | --- | --- |
| Age (Years) |  |  |  |
| mean ± standard deviation (min, max) | 23 ± 3 (18, 34) | 23 ± 4 (18, 35) | **0.060** |
| Age Group (Years) |  |  | **0.159** |
| 18-20 | 104 (25.5%) | 572 (25.7%) |  |
| 21-30 | 286 (70.1%) | 1502 (67.4%) |  |
| 31-35 | 18 (4.4%) | 154 (6.9%) |  |
| Dwelling Type^1^ |  |  | **0.024** |
| Formal | 363 (89.0%) | 1907 (85.6%) |  |
| Informal | 45 (11.0%) | 287 (14.%) |  |
| Type of Living Area |  |  | **0.033** |
| Urban | 343 (84.1%) | 1812 (81.3%) |  |
| Rural | 65 (15.9%) | 382 (17.1%) |  |
| Medical Aid^2^ |  |  | **0.554** |
| Yes | 4 (1.0%) | 33 (1.5%) |  |
| Educational Level |  |  | **0.06** |
| No formal education | 3 (0.7%) | 7 (0.3%) |  |
| Primary education | 225 (55.1%) | 1126 (50.5%) |  |
| Secondary education | 176 (43.1%) | 1081 (48.5%) |  |
| Tertiary education and above | 4 (0.9%) | 14 (0.6%) |  |
| No formal education | 3 (0.7%) | 7 (0.3%) |  |
| Country of Residence |  |  | **<0.001** |
| Malawi | 26 (6.4%) | 131 (5.9%) |  |
| Mozambique | 12 (2.9%) | 33 (1.5%) |  |
| South Africa | 210 (51.5%) | 1564 (70.2%) |  |
| Zambia | 91 (22.3%) | 238 (10.7%) |  |
| Zimbabwe | 69 (16.9%) | 262 (11.8%) |  |
| Had a partner living with HIV |  |  | **0.248** |
| Do not know | 185 (45.3%) | 1050 (47.1%) |  |
| No | 211 (51.7%) | 1139 (51.1%) |  |
| Yes | 12 (2.9%) | 39 (1.8%) |  |
| Any sexually transmitted infection^3^ |  |  | **0.536** |
| Negative | 272 (66.7%) | 1520 (68.2%) |  |
| Positive | 136 (33.3%) | 708 (31.8%) |  |
| Syphilis^4^ |  |  | **0.518** |
| Negative | 398 (97.5%) | 2150 (96.5%) |  |
| Not done/indeterminate | 1 (0.2%) | 5 (0.2%) |  |
| Positive | 9 (2.2%) | 73 (3.3%) |  |
| Trichomonas^5^ |  |  | **0.607** |
| Negative | 361 (88.5%) | 1995 (89.5%) |  |
| Not done/indeterminate | 6 (1.5%) | 40 (1.8%) |  |
| Positive | 41 (10.0%) | 193 (8.7%) |  |
| N. Gonorrhea^6^ |  |  | **0.504** |
| Negative | 378 (92.6%) | 2048 (91.9%) |  |
| Not done/indeterminate | 2 (0.5%) | 25 (1.1%) |  |
| Positive | 28 (6.9%) | 155 (7.0%) |  |
| C. Trachomatis^6^ |  |  | **0.427** |
| Negative | 319 (78.2%) | 1732 (77.7%) |  |
| Not done/indeterminate | 8 (2.0%) | 27 (1.2%) |  |
| Positive | 81 (19.9%) | 469 (21.1%) |  |
| Pregnancy Prevention Method^7^ |  |  | **<0.001** |
| Intrauterine device (IUD) or system (IUS) | 5 (1.2%) | 35 (1.6%) |  |
| Implants | 124 (30.4%) | 458 (20.6%) |  |
| Injectable contraceptives | 204 (50.0%) | 1454 (65.3%) |  |
| Oral contraceptives | 47 (11.5%) | 104 (4.7%) |  |
| Multiple, other | 28 (6.9%) | 167 (7.5%) |  |
| Number of babies alive at birth at study entry |  |  | **0.146** |
| 0 | 95 (23.3%) | 451 (20.6%) |  |
| 1 | 205 (50.2%) | 1067 (48.6%) |  |
| 2 or more | 108 (26.5%) | 1. 0.3%) |  |

**Figure S1:**


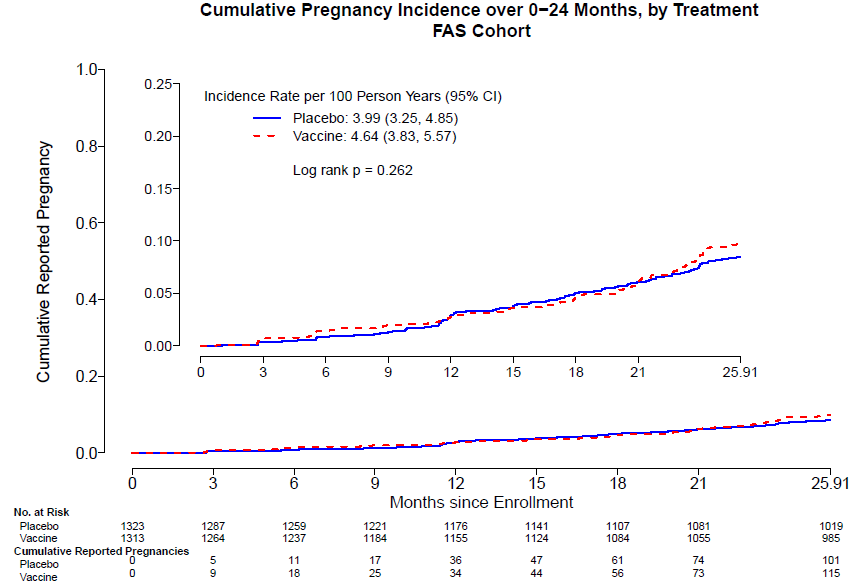


**Figure S1**. Cumulative reported pregnancy incidence, months 0-24, among all participants who received at least one study product administration, stratified by country of enrollment. Data were censored at 25.91 months which includes follow up time for participants who came late for the Month 24 visit. FAS = full analysis set, including all participants who received at least one study product administration.

**Figure S2:**


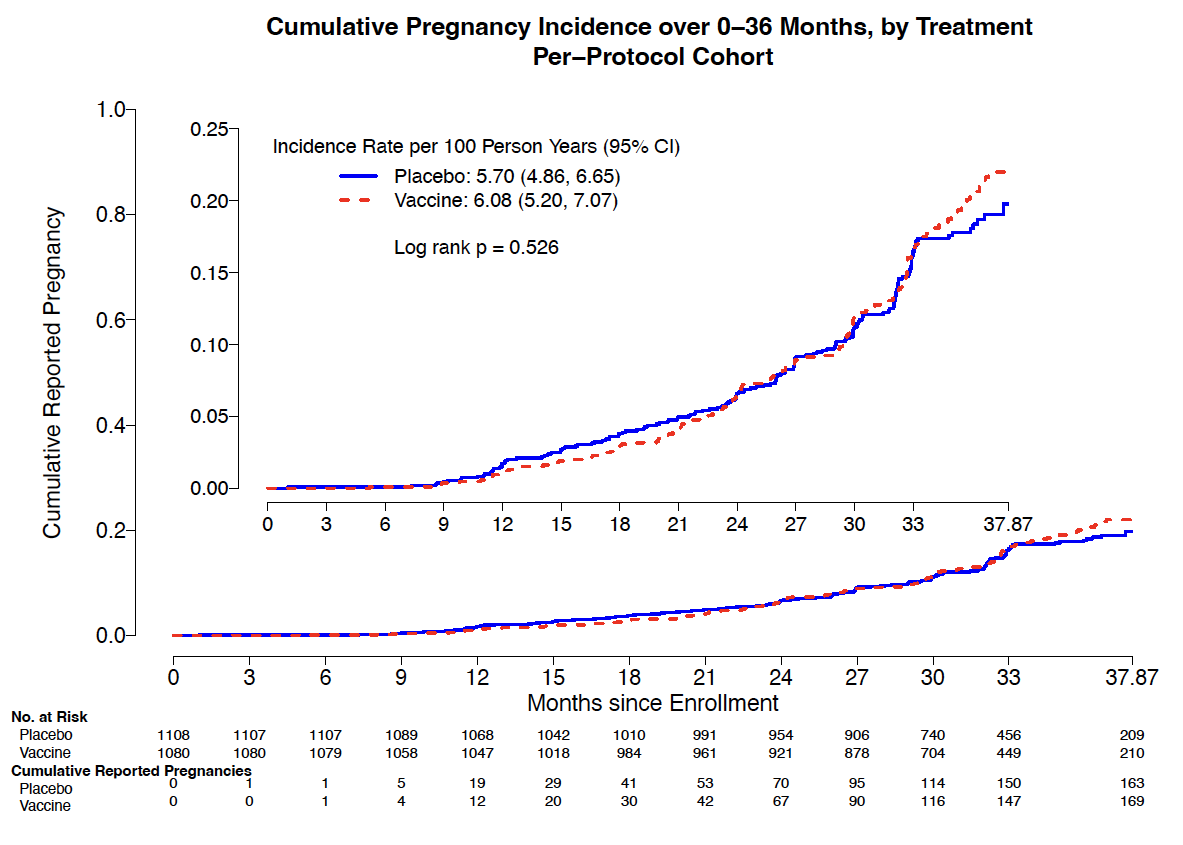


**Figure S2**. Cumulative reported pregnancy incidence, months 0-36, among all participants who received at least one study product administration, stratified by vaccine v. placebo. Data were censored at 37.87 months which includes follow up time for participants who came late for the Month 36 visit.

**Figure S3:**


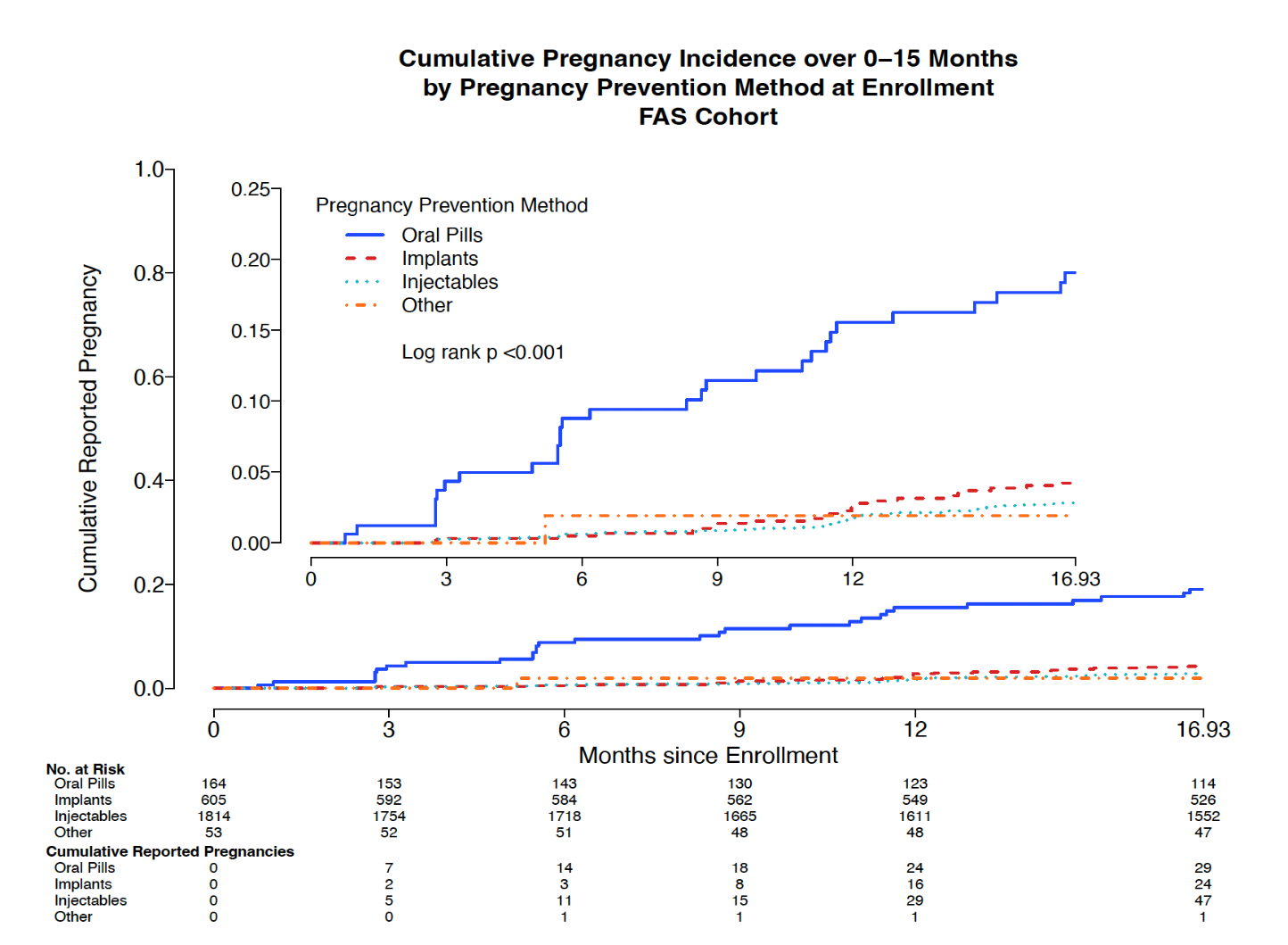


**Figure S3**. Cumulative reported pregnancy incidence, months 0-15, among all participants who received at least one study product administration, stratified by pregnancy prevention method. Pregnancy prevention method was modelled as a time-varying covariate. Data were censored at 16.93 months which includes follow up time for participants who came late for the Month 15 visit. FAS = full analysis set, including all participants who received at least one study product administration.
